# Cartilage Damage Pathological Characteristics of Diabetic Neuropathic Osteoarthropathy

**DOI:** 10.1155/2023/7573165

**Published:** 2023-05-08

**Authors:** Pei-Long Liu, Jia-Yu Diao, Qiong Wang, Huan Liu, Yan Zhang, Jing-Qi Liang, Feng Zhang, Xiao-Jun Liang, Hong-Mou Zhao

**Affiliations:** ^1^Foot and Ankle Surgery Department, Honghui Hospital of Xi'an Jiaotong University, No. 76 Nanguo Road, Xi'an 710054, China; ^2^Cardiovascular Department, Shaanxi Provincial People's Hospital, Xi'an 710068, China; ^3^School of Public Health, Xi'an Jiaotong University, Xi'an 710086, China

## Abstract

**Background:**

Diabetic neuropathic osteoarthropathy (DNOAP) is a rare and easily missed complication for diabetes that leads to increased morbidity and mortality. DNOAP is characterized by progressive destruction of bone and joint, but its pathogenesis remains elusive. We herein aimed to investigate the pathological features and pathogenesis of the cartilages damage in DNOAP patients.

**Methods:**

The articular cartilages of eight patients with DNOAP and eight normal controls were included. Masson staining and safranine O/fixed green staining (S-O) were used to observe the histopathological characteristics of cartilage. The ultrastructure and morphology of chondrocytes were detected by electron microscopy and toluidine blue staining. Chondrocytes were isolated from DNOAP group and control group. The expression of receptor activator of nuclear factor kappaB ligand (RANKL), osteoprotegerin (OPG), interleukin-1 beta (IL-1*β*), interleukin-6 (IL-6), tumor necrosis factor-alpha (TNF-*α*), and Aggrecan protein was evaluated by western blot. Reactive oxygen species (ROS) levels were measured using a 2′,7′-dichlorofluorescin diacetate (DCFH-DA) probe. The percentage of apoptotic cells was determined by flow cytometry (FCM). The chondrocytes were cultured with different glucose concentrations to observe the expression of RANKL and OPG.

**Results:**

Compared with the control group, the DNOAP group showed fewer chondrocytes, subchondral bone hyperplasia, and structural disorder, and a large number of osteoclasts formed in the subchondral bone area. Moreover, mitochondrial and endoplasmic reticulum swellings were observed in the DNOAP chondrocytes. The chromatin was partially broken and concentrated at the edge of nuclear membrane. The ROS fluorescence intensity of chondrocyte in DNOAP group was higher than that in normal control group (28.1 ± 2.3 vs. 11.9 ± 0.7; *P* < 0.05). The expression of RANKL, TNF-*α*, IL-1*β*, and IL-6 protein in DNOAP group was higher than that in normal control group, whereas OPG and Aggrecan protein were lower than that in normal control group (both *P* < 0.05). FCM showed that the apoptotic rate of chondrocyte in DNOAP group was higher than that in normal control group (*P* < 0.05). The RANKL/OPG ratio showed significant upward trend when the concentration of glucose was over than 15 mM.

**Conclusions:**

DNOAP patients tend to have severe destruction of articular cartilage and collapse of organelle structure including mitochondrion and endoplasm reticulum. Indicators of bone metabolism (RANKL and OPG) and inflammatory cytokines (IL-1*β*, IL-6, and TNF-*α*) play an important role in promoting the pathogenesis of DNOAP. The glucose concentration higher than 15 mM made the RANKL/OPG ratio change rapidly.

## 1. Introduction

Diabetic neuropathic osteoarthropathy (DNOAP) was first described in1936 by Jordan. It is a serious complication of diabetes, accounting for 0.8–13.0% of all diabetic patients. In addition, the prevalence of high-risk patients can be as high as 29.0% [1]. As diabetes mellitus has become one of the most common disorders now, there will be an increase in the prevalence of DNOAP. Patients with DNOAP often present with joint subluxations, dislocation, or pathological fractures, which reduce the quality of life and increase the mortality significantly [2, 3]. Accurate diagnosis and appropriate treatment could avoid the delays in patient condition and operation, improve clinical outcomes, and lower the medical costs.

The etiology of DNOAP is multifactorial, and the main theories concerning the origin of DNOAP are like neurovascular, neurotraumatic, and neurobone-inflammatory theories [4]. In the neurovascular theory, it was proposed that hyperactive vaso-autonomic neuropathy resulted in a hyperemic state, which in turn promoted capillary permeability due to increased blood flow. Then, compartmental pressure rose, and deep tissue ischemia occurred. Eventually, these changes damaged ligaments and tendons of the foot and ankle. In addition, abundant blood flow increased the delivery of monocytes and osteoclasts, which enhanced bone resorption [5, 6]. In the neurotraumatic theory, acute\subacute\repetitive traumas were assumed to be the causative factor for DNOAP [7]. Various traumas could trigger a cascade of inflammatory events and led to the acute release of pro-inflammatory cytokines, interleukin family [interleukin-1 beta (IL-1*β*) and interleukin-6 (IL-6)], and tumor necrosis factor-alpha (TNF-*α*). In addition, they activated the receptor activator of nuclear factor kappaB ligand (RANKL)–osteoprotegerin (OPG) system to regulate bone turnover. The RANKL/OPG system is a pivotal mediator in bone metabolism. RANKL is a member of the TNF superfamily and plays vital role in promoting osteoclast formation. It regulates osteoclast differentiation and promotes osteoclastogenesis and bone resorption by activating the RANK. OPG is a cytokine synthesized by activated osteoblasts, commonly known as the “osteoclastogenesis inhibitory factor,” and it is the competitive protein of RANKL. It has been proposed that RANKL/OPG axis may be a central link in the development of DNOAP with persistent inflammation state [8, 9].

The pathogenesis of DNOAP, however, remains largely unknown. In addition new prospects for studying DNOAP are constantly emerging. The investigation of DNOAP pointed out that inflammatory markers and the dynamics of bone metabolism were involved in the pathological process of it [10–12]. Among them, the increase of pro-inflammatory cytokines TNF-*α*, IL-6, and IL-1*β* in serum of DNOAP suffers has been reported multiple times [10, 13]. In addition, Jeffcoate proposed that the pathway of receptor activator of nuclear factor kappaB (RANK), its ligand RANKL, and OPG played a role in disease progression of Charcot neuroarthropathy (CN) firstly in 2004 [14].

The RANKL/OPG axis is a key mediator, which has been used to evaluate osteoclastogenesis and osteolytic processes in numerous diseases, such as rheumatoid arthritis, osteoarthritis, and bone tumors. In addition, the expression of RANKL was increased in diabetes caused by oxidative stress and inflammation state [15]. Furthermore, La Fontaine et al. [16] found that the number of bone trabeculae in patients with DNOAP was significantly reduced, and the structure was disordered. However, there is no report on the pathological changes of cartilage from DNOAP patients.

In this study, articular cartilage specimens and chondrocytes were used to explore the pathological characteristics and molecular mechanism of DNOAP patients, so as to open novel avenues for clinical prevention and treatment.

## 2. Materials and Methods

### 2.1. Clinical Samples

From March 2017 to June 2018, articular cartilage specimens were collected from eight clinically confirmed DNOAP patients, and the articular cartilage of matched joints from eight amputation patients without underlying disease was as control group.

Inclusion criteria for DNOAP patients are as follows: (1) DNOAP was definitely diagnosed according to the diabetes mellitus history, clinical, and imaging manifestations; (2) type I–IIIA according to Brodsky classification [17]; (3) phase III judged by Eichenholtz classification system [18]; and (4) patients underwent middle or posterior foot fusion surgery. Exclusion criteria are as follows: (1) patients with peripheral arterial disease; (2) active infection and ulcer; and (3) unclear diagnosis of DNOAP. The eight patients with DNOAP were three males and five females, aged 20–66 (55.7 ± 3.8) years, diabetes mellitus duration of 6–14 (11.7 ± 3.1) years, and DNOAP duration of 5–13 (7.3 ± 4.3) months. Two cases were Brodsky type I, four cases were type II, and two cases were type IIIA.

Enrolled controls were patients without diabetes and peripheral neuropathy who underwent amputation due to traffic accident or serious trauma. Exclusion criteria are as follows: (1) osteoarthritis, rheumatoid arthritis, and other degenerative joint disease; and (2) open injury or contamination of affected joints. Eight cases of traffic accident or severe injury amputation were recruited as the controls, including four males and four females, aged 19–65 (57.6 ± 3.7) years old.

The articular cartilages of tibiotalar joint, subtalar joint, and talonavicular joint were taken from two groups. The use of human samples was approved by the Ethical Committee of Honghui Hospital of Xi'an Jiaotong University (approval No. 201702003), and informed consent was obtained from each participant.

### 2.2. Pathological Examination

The articular cartilage biopsies from donors of DNOAP group and controls were fixed in 10% formaldehyde for 24 hours, decalcified by 15% neutral ethylenediaminetetraacetic acid disodium salt (EDTA-2Na) for 15 days, then dehydrated by alcohol gradient of different concentrations and embedded with paraffin. The paraffin-embedded specimens were cut into 5 *μ*m consecutively. Then, the tissue sections were stained with Masson's Trichrome Stain Kit and Modified Safranine O-Fast Green FCF Cartilage Stain Kit (Beijing Solarbio Science & Technology Co., Ltd., Beijing, China), according to the manufacturer's instructions. Finally, the pathological changes of cartilage, calcified layer, and subchondral bone were observed directly under the optical microscope.

### 2.3. Transmission Electron Microscopy

The fresh cartilage specimens of DNOAP group and controls were fixed with 2.5% glutaraldehyde at 4°C in a volume of 1 mm^3^ overnight. The next day, the cartilage samples were rinsed with ddH_2_O, fixed with 1% osmium acid for 1 hour, stained with 2% uranium acetate for 30 minutes, and dehydrated gradiently with 50%, 70%, 90%, and 100% ethanol and 100% acetone. After infiltration, embedding, and polymerization, the sections were sliced into a thickness of 70 nm by ultramicrotome and then stained with uranium acetate lead citrate. The ultrastructure of organelles in chondrocytes was observed under HITACHIH-7650 transmission electron microscope (Hitachi, Tokyo, Japan).

### 2.4. Toluidine Blue Staining

To investigate the cartilage morphology to chondrocytes in DNOAP group and controls, toluidine blue dye was applied to compare their difference. The fresh cartilage specimens of DNOAP group and controls were fixed in 4% paraformaldehyde for 20 minutes and then stained with toluidine blue dye solution (APPLYGEN, Toluidine Blue O Cartilage Stain Solution B1104) for around 30 minutes at room temperature. Images were captured using fluorescence microscopy (Leica DMI 3000 M, German).

### 2.5. Chondrocyte Maintenance

The cartilage samples were segmented into 3–5 mm^3^ pieces and washed into the phosphate buffer solution (PBS) containing penicillin (100 U/L) and streptomycin (100 mg/L). Then, the small pieces were digested with 0.25% trypsin at room temperature for 15–20 minutes and centrifuged at 1000×*g* for 5 minutes. After removing the supernatant, the deposit was washed with PBS for three times. Next, the deposit was digested at 37°C for 8–10 hours with 0.2% Collagenase Type II (C2-BIOC, MilliporeSigma, Burlington, MA, USA) in a constant temperature shaker. After filtration by aseptic cell sieves, the cells were washed and collected through centrifugation (1000×*g*, 10 minutes). The chondrocytes were cultured with Dulbecco's Modified Eagle's Medium (DMEM) medium (Invitrogen, USA) supplemented with 10% fetal bovine serum (Thermo Fisher Scientific, USA) at 37°C in a humidified atmosphere containing 5% CO_2_.

### 2.6. Detection of Reactive Oxygen Species in Chondrocytes

Reactive oxygen species (ROS) production was detected with a Reactive Oxygen Species Assay Kit (Shanghai Beyotime Biotechnology Co., Ltd., Shanghai, China). Inoculate the chondrocytes into 6-well plate (1 × 10^6^ cells per well), and 3 auxiliary holes were set for each group. When the cell fusion rate reached 70–80%, the culture medium was removed, and 2′,7′-dichlorofluorescin diacetate (DCFH-DA) with the concentration of 10 *μ*mol/L was added into each well, and then the plate was placed in dark for 20 minutes at 37°C. Wash the cells with serum-free DMEM for three times. ROS were measured by fluorescence microscope at an excitation wavelength of 485 nm and an emission of 525 nm. The intensity of each group was analyzed by the Image Pro Plus image analysis software.

### 2.7. Western Blotting

Protein concentration was detected using Pierce BCA Protein Assay Kit (Thermo Fisher Scientific, Waltham, MA, USA) in accordance with the manufacturer's instructions. Total proteins were electrophoretically separated on sodium dodecyl sulfate polyacrylamide gels (8–15%) according to the molecular size of the target protein and were subsequently transferred onto polyvinylidene difluoride membranes. After being blocked with 5% skim milk, the membranes were incubated at 4°C overnight with the following primary antibodies: anti-RANKL, anti-OPG, anti-IL-1*β*, anti-IL-6, anti-TNF-*α*, and anti-*β*-actin (Proteintech, Chicago, IL, USA).

Then, the membranes were washed thoroughly and incubated with secondary antibodies (1 : 2000 anti-mouse/rabbit, Santa Cruz Biotechnology, Dallas, TX, USA) at room temperature for 2 hours. The signals were visualized using the enhanced chemiluminescence method (Immobilon Western Chemiluminescent HRP Substrate, MilliporeSigma). The samples were analyzed in duplicates, and the experiment was performed three times.

### 2.8. Detection of Apoptosis by Flow Cytometry

The percentage of apoptotic cells was ascertained through Annexin V-Fluorescein Isothiocyanate/Propidium Iodide (FITC/PI) apoptosis detection kit (Beijing Sizhengbai Biotechnology Co., Ltd., Beijing, China).

Cells were prepared with the concentration of 1 × 10^6^/ml in 10% bovine serum albumin, according to the manufacturer's instructions. After cultured in the incubator with 5% CO_2_ at 37°C for 24 hours, the cells were collected in a 10 ml centrifuge tube, centrifuged at 1000×*g* for 5 minutes and washed with precooled PBS twice. The Annexin V-FITC/PI and PI (100 *μ*g/ml) working solution were added into cells, and stained for 15 minutes at room temperature in dark. Flow cytometry (FCM) was used to detect the apoptosis rate of cells, and the Cell Quest software was used to obtain and analyze parameters. The experiment was repeated three times.

### 2.9. High Glucose Induced Expression of RANKL and OPG in Chondrocytes

Hyperglycemia is a common feature of DNAOP patients; thus, we simulated anomalous level of blood sugar in vitro by treating normal chondrocytes with a wide range of glucose concentrations (5, 10, 15, 20, 25, and 33 mM) for 24 hours. Western blot analysis was used to detect the expression of RANKL and OPG in chondrocytes.

### 2.10. Statistical Methods

Statistical analyses were performed with the SPSS 19.0 (SPSS Inc., Chicago, IL, USA). Data from cell experiments were analyzed using unpaired Student's *t*-tests, and images based on the statistical analyses were made in the GraphPad Prism 5.0 (GraphPad Software Inc, La Jolla, CA, USA). All hypothetical tests were two-sided, and *P*-values less than 0.05 were considered statistically significant in all tests.

## 3. Results

### 3.1. Histopathological Characterization of Articular Cartilage

**Figure 1:**
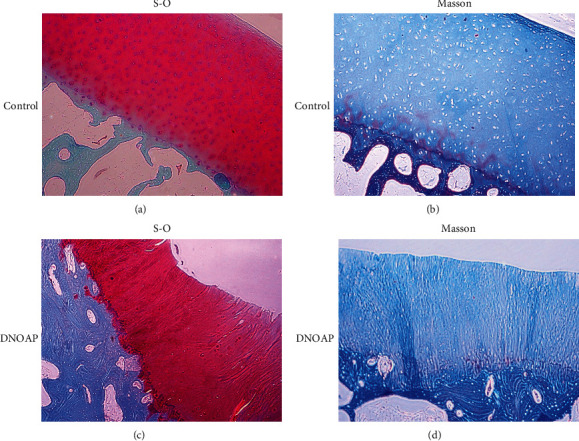
Pathological changes of cartilage in each group under S-O staining and Masson staining (100×). Representative S-O staining of cartilage in the control group (a) and DNOAP group (b). Representative Masson staining of cartilage in the control group (c) and DNOAP group (d). S-O, Modified Safranine O-Fast Green FCF Cartilage Stain; Masson, Masson's trichrome stain.

Changes in articular cartilage were explored under the optical microscope. In the articular cartilage from the control group, chondrocytes were located in cartilage lacuna, cartilage matrix was stained evenly, and subchondral bone was arranged orderly (Figures 1(a) and 1(b)). However, in the tissues of DNOAP patients, S-O staining showed that the continuity of the superficial cartilage was interrupted, the vertical fracture entered into the deep layer of cartilage. The chondrocytes around the fracture were ruptured, and the matrix was light stained. Subchondral bone plates and trabeculae were reduced, and osteoclasts aggregation was observed. The subchondral bone shows the characteristics of reactive bone, and the structure is disordered (Figure 1(c)). Masson's trichrome staining showed that hyaline cartilage was arranged in a cordlike arrangement, and the chondrocytes and osteocytes were decreased. In addition, subchondral bone hyperplasia, structural disorder, and cavities were observed (Figure 1(d)). The histopathological findings of all DNAOP patients were consistent.

### 3.2. Ultrastructure and Morphology of Chondrocytes

**Figure 2:**
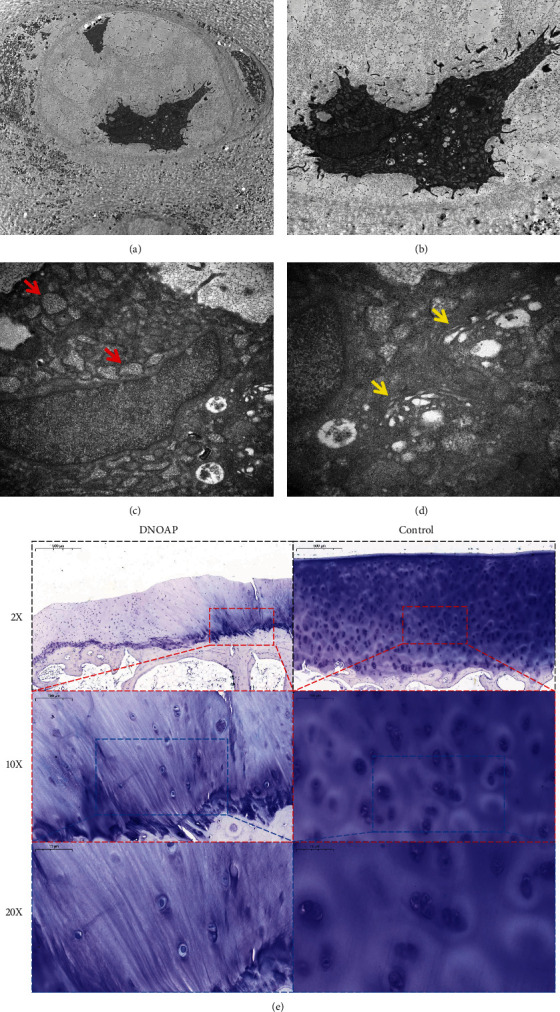
Ultrastructure of chondrocytes in DNOAP group and Toluidine blue staining of chondrocytes in two groups. (a) The chondrocytes are located in the cartilage vortex (4x) under transmission electron microscope; (b–d) they were amplified by 10×, 30× and 50×, respectively, showing the formation of a large number of vesicles in the cytoplasm, significant mitochondrial swelling, intima rupture, mitochondrial crest arrangement disorder (c, red arrow), and obvious swelling of the Golgi apparatus and endoplasmic reticulum (d, yellow arrow). (e) Representative pictures of chondrocytes of toluidine blue staining in DNOAP group and control group.

The integrity of organelles is the primary condition for cellular homeostasis. Next, we observed the ultrastructure of chondrocytes by transmission electron microscope. In the chondrocytes from DNOAP group, a large number of vesicles in the cytoplasm and swollen mitochondria were found, some mitochondrial membranes were incomplete, and the arrangement of mitochondrial cristae was disordered. Furthermore, severe expansion of the endoplasmic reticulum and Golgi apparatus was scattered in the cytosol. The nucleus became larger, and the chromatin was partially broken, concentrated, and gathered at the edge of nuclear membrane (Figures 2(a), 2(b), 2(c), and 2(d)). These changes induce limited energy sources required by the cells and metabolic imbalance. In addition, toluidine blue staining in cartilage specimens showed that the articular surface is flat, the tidemark is complete, and the chondrocytes morphology is normal in the control group. However, in the DNOAP group, subchondral bone hyperplasia and tidemark destruction were observed. Chondrocytes were morphologically altered and decreased in number (Figure 2(e)).

### 3.3. ROS Increased in Chondrocytes of the DNOAP Group

**Figure 3:**
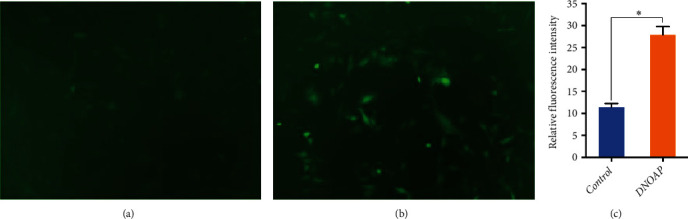
The level of ROS increased in chondrocytes of the DNOAP group. Representative images were taken in control group (a) and DNOAP group (b) via fluorescence microscope (×100). (c) The result depicts the comparative analysis of ROS levels in chondrocytes of two groups. The error bars presented as mean ± standard error of mean (SEM) with analysis of unpaired Student's *t*-test. ∗*P* < 0.05, compared with control group.

The mitochondrion supplies energy with cells and produces Adenosine Triphosphate (ATP). The production of ATP depends on oxidative phosphorylation, and ROS is the main by-product of this process. ROS are highly reactive molecules that provide the normal signals in various cell types. However, the accumulation of ROS leads to oxidative damage of biomolecules, which further causes oxidative stress and even cell death. Thus, we detected the level of ROS in chondrocytes by fluorescence microscope, and found that the ROS fluorescence intensity of cells originated from DNOAP group was obviously higher than the cells from the controls (28.1 ± 2.3 vs. 11.9 ± 0.7, *t* = 19.059; *P* < 0.05; Figure 3).

### 3.4. Apoptosis Increased in Chondrocytes Originated from DNOAP Patients

**Figure 4:**
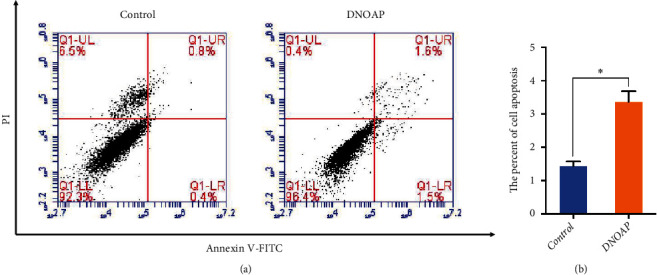
The percentage of apoptotic chondrocytes increased in the DNOAP group. (a) Representative images of FCM using Annexin V-FITC and PI staining. (b) Column bar graph showing a dramatically bigger early and late apoptosis ratio in cells of DNOAP patients than the controls. Each group was independently repeated three times, and 3000 cells were calculated. The error bars presented as mean ± SEM with analysis of unpaired Student's *t*-test. ∗*P* < 0.05, compared with control group.

We then compared the cell apoptosis between the DNOAP group and the control group. FCM analysis demonstrated that the proportion of apoptotic chondrocytes was around 2.6 times higher in the DNAOP group than in the control group (3.3 ± 0.2% vs. 1.2 ± 0.1%, *P* < 0.05; Figure 4).

### 3.5. The Expression of RANKL, OPG, Aggrecan, and Inflammatory Cytokines

**Figure 5:**
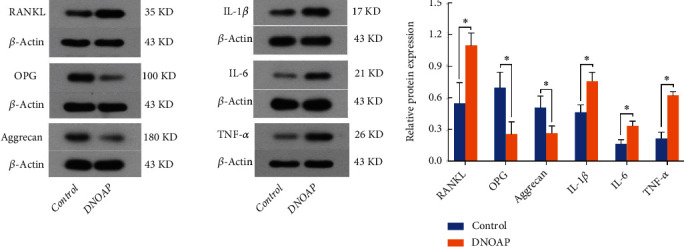
The protein expression of RANKL, OPG, Aggrecan, IL-1*β*, IL-6, and TNF-*α* in cartilage specimens of DNOAP and control group. Representative western blot bands and analysis in samples of DNOAP and control group. *β*-Actin was used as a reference for calculating the relative protein expression. The error bars presented as mean ± SEM with analysis of unpaired Student's *t*-test. ∗*P* < 0.05, compared with control group.

Western blot analyses were performed on total protein extracted from articular cartilage specimens of DNOAP patients and control participants. It demonstrated that the tissues of DNOAP group expressed higher levels of RANKL, IL-1*β*, IL-6, and TNF-*α* and lower levels of OPG and Aggrecan than that of normal group, respectively (*P* < 0.05; Figure 5). The comparison results of each pair of samples and age-matched controls were consistent. In addition, representative images of each index detected in our study were shown in Figure 5.

### 3.6. High Glucose Induced the Change of RANKL/OPG Ratio in Chondrocytes

**Figure 6:**
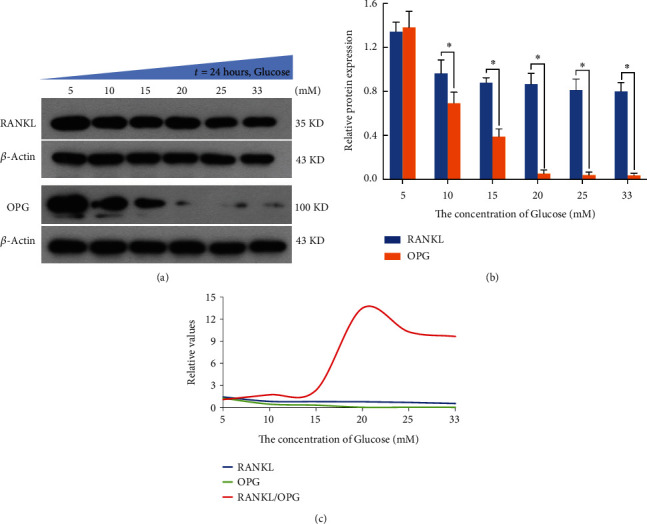
The expression of RANKL, OPG, and RANKL–OPG ratio changed under various concentration of glucose. Representative western blot bands (a) and analysis (b and c) of RANKL and OPG in chondrocytes under different concentrations of glucose. *β*-Actin was used as a reference for calculating the relative protein expression. Each group was repeated at least three times. The error bars presented as mean ± SEM with analysis of unpaired Student's *t*-test. ∗*P* < 0.05, compared with the control group.

Western blot analyses demonstrated that the expression of RANKL decreased slightly with glucose concentration increase of DMEM medium. However, a sharp decline in OPG was observed, and when the concentration of glucose was or over than 20 mM, the expression of OPG was almost undetectable. Then, we found that the ratio of RANKL/OPG remained near 1 : 1 when the cells were cultured with 5 mM glucose, but the ratio showed significant upward trend when the concentration of glucose was over than 15 mM. The ratio reached the peak at 20 mM of glucose, and then decreased slightly and almost remained at the same level (Figure 6).

## 4. Discussion

Neuroarthropathy, also known as Charcot's joint, refers to the progressive, painless, and noninfective destructive disease of bone and joint caused by neuropathy. In 1886, Charcot first reported the bone and joint lesions of patients with spinal tuberculosis comprehensively. In 1936, Jordan found the relationship between diabetes and neuroarthropathy, and proposed the concept of DNOAP for the first time [19]. However, up to now, the pathogenesis of DNOAP have remained unclear. Limited previous studies focused on the changes of synovium and bone trabeculae. In this study, we detected the pathological changes of cartilage first.

There were a large number of chondrocytes disintegrated in the cartilage tissue of DNOAP patients, osteoclasts aggregation, and subchondral bone remodelling in subchondral bone area. In addition, all of the changes of cartilage above were not found in osteoarthritis or rheumatoid arthritis [20]. Osteoclasts are closely related to bone resorption [21]. Activation and aggregation of osteoclasts will cause bone resorption and destruction, and lead to development of DNOAP. In addition, we observed the ultrastructural features of chondrocytes originated from DNOAP patients with transmission electron microscope, and found mitochondrial swelling and disordered arrangement of mitochondrial cristae, and numerous vacuoles in the endoplasmic reticulum and Golgi apparatus. Moreover, the apoptotic rate of chondrocytes in DNOAP group was higher than that in control group. The activity of mitochondrial and endoplasmic reticulum is closely related to the cell viability [22]. Structural damage of mitochondria and endoplasmic reticulum will lead to the changes in cellular metabolism, even the programmed death of chondrocytes, namely apoptosis, and finally induced to the destruction of cartilage tissue structure and matrix.

ROS are the main substance that causes oxidative stress damage in tissues, which plays an important role in bone remodelling by stimulating RANKL and inhibiting OPG expression [23, 24]. ROS had a negative effect on osteoblastic differentiation [25]. On the contrary, osteoclast activity was directly stimulated by ROS [26]. Verzijl et al. found that the deposition of advanced glycation end products and ROS was increased in chondrocytes exposed to high-glucose [27].

Mitochondria are one of important organelles that maintain cell metabolism, and they provide most of the energy (ATP) needed by the cells. Moreover, mitochondria are involved in various biological functions (proliferation, differentiation, ions homeostasis, and cell fate). The main by-product of ATP production is ROS. It has been reported that about 90% ROS is generated by the process. Normal physiological processes can produce ROS, but the abnormal increase of ROS or mitochondria dysregulation is associated with many diseases. Currently, it has been known that energy imbalance was linked to neurological disorders, cancers, and metabolic diseases. The destruction of organelle integrity leads to the imbalance of intracellular environment and the barrier of information transmission between cells [28]. Gap junctions, a form of intercellular connections, have been implicated in the pathogenesis of diabetes and its complications. Gap junction proteins are the basis for these special connections, allowing bidirectional flow of ions and small molecules. They include the connexins family, which were named based on their molecular weight [29]. It has been found that the expression of connexins was correlated with blood glucose concentration [30]. Take Cx-43 for example, several studies demonstrated that it played a protective role in the progression of diabetes. The Cx-43 levels increased with the increase of blood glucose, then ion transfers, and cell–cell communication were more active found in nephropathy study. Another study also showed that Cx-43 could weaken ROS level in diabetic patients by activating Nrf2/ARE signal pathway [31]. In our study, we found the morphological changes of mitochondria and other organelles, and monitored the increase of ROS level. The known role of gap junction proteins provides an important direction for our further mechanistic studies.

In addition, we found that the production of ROS in DNOAP chondrocytes was more than the healthy controls, accompanied by higher level of RANKL and lower of OPG. These findings suggested that ROS induced by hyperglycemia may stimulate osteoclast activation by regulating RANKL/OPG pathway in DNOAP patients. RANKL/OPG system, as an important regulatory axis of bone turnover, plays an important role in the activation, aggregation, and function of osteoclasts. RANKL belongs to the TNF superfamily and has the ability to promote osteoclast formation. Its main role is to activate the RANK, which regulates osteoclast differentiation, promotes osteoclastogenesis, and bone resorption [32]. OPG, the soluble decoy receptor of RANKL, is a cytokine synthesized by activated osteoblasts, commonly known as the “osteoclastogenesis inhibitory factor.” OPG antagonizes the RANKL–RANK interactions on the surfaces of osteoclast progenitors and blocks the resultant downstream osteoclastogenic cascade [33]. In a nutshell, osteoclast activity is likely to depend, at least in part, on the relative balance of RANKL and OPG. In general, abnormal RANKL : OPG ratio predicts pathological states and can lead to an uncontrolled loss of bone mass [34, 35].

Under stable bone metabolism conditions, the secretion and expression of RANKL and OPG are dynamically balanced to maintain the homeostasis of osteogenesis and osteoclast. When the expression of RANKL increased and OPG decreased, osteoclasts would aggregate and activate, leading to bone destruction [36, 37]. In the present study, we found that the expression of RANKL in DNOAP group was significantly higher than that in normal control group, whereas an opposite trend was observed for the OPG expression in two groups. These results suggested that the RANKL/OPG pathway may be involved in the pathological changes of cartilage in the patients with DNOAP. Previous studies failed to conclude the relationship between the RANKL/OPG pathway and the progression of DNOAP in peripheral blood [10]. Thus, the change of RANKL and OPG expression in the local cartilage lesion would be a significant discovery in the study of DNOAP.

Abnormal inflammatory markers are a common feature in DNOAP patients [38, 39]. Generally, pro-inflammatory cytokines regulate the inflammatory response and have dynamic interactions with metabolites that could mediate the bone turnover [40]. Kwan Tat et al. reported that the expression of RANKL was increased and OPG decreased in osteoarthritis chondrocytes under the stimulation of IL-1*β*, TNF-*α*, and PGE2 [41]. In our study, it was found that the expression of IL-1*β*, IL-6, and TNF-*α* in DNOAP chondrocytes was significantly higher than that in the control group. Previous studies have reached similar conclusions [13, 42]. Therefore, we speculated that the effects of these inflammatory indicators on DNOAP may partly depend on the biochemical activation of the RANKL/OPG signaling pathway.

Persistent hyperglycemia is a common clinical manifestation in patients with DNOAP [43]. We treated normal chondrocytes with glucose in different concentrations and found that the expression of RANKL decreased slightly and OPG sharply with glucose concentration increase. Notably, RANKL/OPG ratio showed obvious upward trend when the concentration of glucose was from 15 to 20 mM. Subsequently, the ratio decreased slightly and almost remained at the same level. Thus, the findings suggested that 15 mM may be a threshold for the imbalance of chondrocyte metabolism, and may also be an important node that triggers the damage of cartilage and even the occurrence of DNOAP.

DNOAP is characterized by inflammatory conditions and osseous resorptive destruction. For a long time, studies have been carried out to prevent the progression of DNOAP and improve patient outcomes. So far, there are some drugs for DNOAP at the acute stage, but their clinical effects are still controversial. leukemia inhibitory factor (LIF) is secreted mainly by osteoclasts, and the main function of it is to inhibit sclerostin. Sclerostin can further negatively regulate the Wnt/*β*-catenin signal pathway. The addition of LIF resulted in a decrease in sclerostin, followed by activation of Wnt signaling and increased bone formation. Therefore, it is reasonable to speculate that LIF may play a role in inhibiting the progression of DNOAP [44]. Bisphosphonates (BPs) are common drugs used to inhibit bone resorption, prevent fractures, and improve bone quality. They decreased the osteoclast activity and have a strong inhibition of bone resorption. The increase of osteoclast activity is the key to the pathogenesis of DNOAP. Anti-resorptive agents that target osteoclastogenesis are logical therapeutic drugs for the cessation of resorption in active DNOAP. It was reported that BPs like pamidronate, alendronate, and zoledronate have been evaluated in treating active DNOAP [45, 46]. However, their effectiveness is controversial. Parathyroid hormone and its analogues are a new class of anabolic therapy for severe osteoporosis, and they can improve bone microstructure. However, whether it really makes sense in the treatment of DNOAP has yet to be determined. Calcitonin targets osteoclasts and acts through regulating the RANKL pathway [47]. It showed that there is significantly reduction of bone turnover markers in patients with DNOAP [48]. In addition, it was concluded that the treatment of acute Charcot neuroarthropathy by calcitonin could be an effective method to prevent bone resorption and disease development. Another drug that might be effective in DNAOP treatment is RANKL antibody. Denosumab (DMB) is a fully human monoclonal anti-RANK ligand (anti-RANKL) antibody that inhibits the differentiation and maturation of osteoclasts by the binding of RANKL to RANK. Thus, DMB can powerfully suppress the osteolytic function of osteoclasts and increase bone mineral density in patients with osteoporosis and bone resorptive disease, such as CN [49]. Studies have shown that DMB injection shorten the acute phase of DNOAP [50, 51]. However, the evidence is limited, and the large sample, randomized, controlled studies are still needed.

In summary, there is no clear and effective drug to stop the progression of the disease. Moreover, according to our clinical observation, the condition of each patient is very heterogeneous. In this study, we found the pathological changes of cartilage in patients with DNOAP, and analyse the pathological characters and molecular pathogenesis of abnormal cartilages in DNOAP. The origin of cartilage damage is a hot topic in current studies. It may provide new clues in the prevention or early treatment of DNOAP.

Furthermore, some new biomaterials should be considering for treating DNOAP. Take hyaluronic acid (HA) for example, it has the characteristics of biocompatibility, biodegradability, and non-toxicity, and was the main component of extracellular matrices and synovial fluid. It plays a variety of biological functions in angiogenesis, inflammation, and development. After hydrogel formation, HA is readily converted into elastic sheets for use in biomedical applications. HA used hydrogels (HA-hydrogels) have been used as vectors in cellular communication and medication research. It has been found that HA-hydrogels could enhance the viability and differentiation of Mesenchymal Stem Cells (MSCs) [52], and deliver them to the full-thickness defects in the patellar groove of rabbit femoral articular cartilage, and the defects were repaired completely three months later [53]. In addition, HA-hydrogels could deliver molecule and control the half-life of many cytokines [54]. In tissue regeneration and repair, HA-hydrogels were considered to be a good medium. For example, the injection of HA-hydrogels promoted articular cartilage repair [55]. In present study, we mainly focus on the pathological characters and molecular pathogenesis of cartilages in DNOAP patients, and the results may provide ideas for obtaining specific markers. Therefore, HA used hydrogels have the potential to control disease progression by blocking abnormal indictors in this study or serve as a vehicle for our future research. In addition, we declare that some of this manuscript is as reported in the preprint [56]. And, some of the contents were included in the form of abstract in AOFAS annual meeting 2020 [57].

## 5. Conclusions

In this study, we reported the pathological features of cartilage and ultrastructural changes of chondrocytes in DNOAP patients for the first time. Indicators of bone metabolism (RANKL and OPG) and inflammatory cytokines (IL-1*β*, IL-6, and TNF-*α*) play an important role in promoting the pathogenesis of DNOAP. The glucose concentration higher than 15 mM made the RANKL/OPG ratio changed rapidly. These results settle a foundation for the further study on the pathogenesis of DNOAP. The broader involvement and clinical relevance of cartilage in the pathogenesis of DNOAP will be the focus of future investigations.

## Data Availability

Data supporting this research article are available from the corresponding author or first author on reasonable request.

## Abbreviations

DNOAP: Diabetic neuropathic osteoarthropathy
IL-1*β*: Interleukin-1*β*
IL-6: Interleukin-6
OPG: Osteoprotegerin
RANK: Receptor activator of nuclear factor kappaB
RANKL: Receptor activator of nuclear factor kappaB ligand
ROS: Reactive oxygen species
S-O: Safranine O/fixed green staining
TNF-*α*: Tumor necrosis factor-*α*.
